# MRI-based visual and haptic catheter feedback: simulating a novel system's contribution to efficient and safe MRI-guided cardiac electrophysiology procedures

**DOI:** 10.1186/1532-429X-16-S1-O50

**Published:** 2014-01-16

**Authors:** Ka-Wai Kwok, Yue Chen, Thomas CP Chau, Wayne Luk, Kent Ronald Nilsson, Ehud J Schmidt, Zion T Tse

**Affiliations:** 1 College of Engineering, University of Georgia, Athens, Georgia, USA; 2 Computing, Imperial College London, London, UK; 3 Athens Regional Medical Center, University of Georgia, Athens, Georgia, USA; 4 Radiology, Brigham and Women's Hospital, Harvard, Boston, Massachusetts, USA

## Background

MRI-guided Electrophysiology (EP) procedures integrate real-time MRI images with catheter position during Radiofrequency Ablation (RFA) of arrhythmias [1]. Using simultaneous MR catheter tracking and imaging [2], this technology can both guide catheter manipulation and provide dynamic assessment of lesion efficacy [3]. Despite advances in MRI-guided EP, maneuvering catheters to the desired location and ensuring appropriate tissue contact is still challenging inside an MRI due to two issues: (1) inconsistent catheter-tissue contact force (CTCF); and (2) visual-motor disorientation arising from differences between manipulation of the catheter's proximal controlling handle and visualization of the catheter-tissue interface. Both issues can increase the risk of cardiac perforation during catheter manipulation. We hypothesized that a technique based on MR imaging to generate force and vibrotactile alarms, as well as the presentation of a reproducible endoscopic view to the catheter operator, could facilitate precise application of RF energy, thereby increasing efficacy and reducing complications.

## Methods

**Figure 1:**
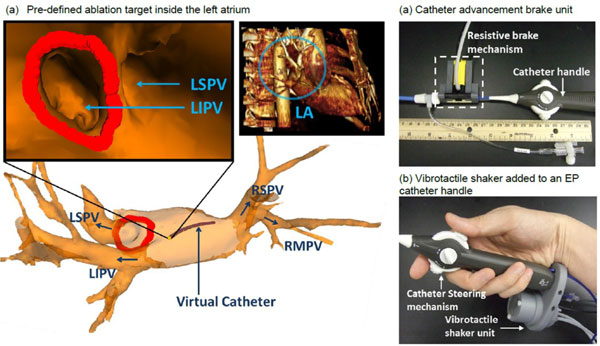
**(a) Catheter endoscopic view visualizing the left superior pulmonary vein (LSPV) and left interior pulmonary vein (LIPV) with predefined RAF targets (in red)**. The left atrium (LA) model (in blue circle) obtained by a 3-D rendering volume; (b-c) Catheter guidance interface with Catheter-Tissue Contact Force alarms.

**Figure 2:**
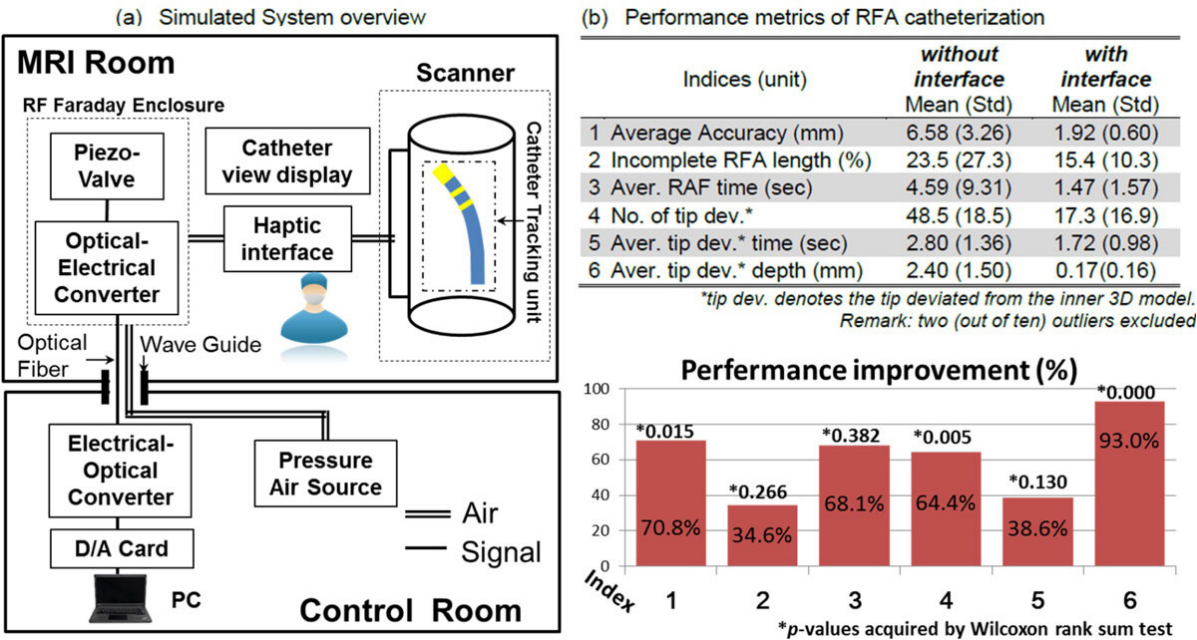
**(a) Simulated System Overview; (b) Performance metrics of a simulated EP RFA procedure performed by ten EP-untrained volunteers**.

Catheter position and cardiovascular structure were updated from MR images (Figure 1a, upper-right), and the magnitude of CTCF was computed using a graphics processing unit (GPU). A collaborative control strategy, Dynamic Active Constraints (DACs) [4], then rendered CTCF alarms to the catheter operator (Figure 1b-c) in the form of: (i) resistive force against excessive advancement of catheter into tissue beyond the imaging model; and (ii) catheter handle vibratory feedback indicating tissue proximity to the RFA targets. The CTCF alarm signals were generated using MR-conditional pneumatic catheter braking and vibrotactile units placed on the catheter's handle, and operated with a 30psi pressure. A virtual camera view (Figure 1a, upper-left) was reconstructed at the catheter tip to provide an endoscopic visualization of the 3-dimensional MRI cardiovascular model. An overview of the complete proposed system is included (Figure 2a).

## Results

Ten volunteers without training in EP procedures were recruited to participate in a simulated RFA procedure to evaluate the performance with and without the proposed endoscopic view and haptic interface. RFA targets were pre-defined at the left pulmonary veins inside a left atrium model reconstructed from the preoperative image data from a cardiac patient (Figure 1a). The subjects were allowed to manipulate the catheter so as to locate the virtual catheter tip in the targets, followed by the performance of ablations at multiple locations around the pulmonary vein ostium. Six performance indices with/without the use of the proposed interface are shown in Figure 2b. On average, subjects demonstrated a 61.6% (σ = 22%) improvement in terms of RFA accuracy, efficiency and safety.

## Conclusions

The proposed image-based catheter haptic guidance and endoscopic view improved RFA procedural time and accuracy, and reduced the risk of perforation.
